# Comparative genomic analysis of *Mycoplasma agalactiae* strain GM139 highlights unique surface architecture and pathogenic determinants

**DOI:** 10.1186/s13567-025-01531-x

**Published:** 2025-05-24

**Authors:** Rohini Chopra-Dewasthaly, Katja Sommer, Maysa Santos Barbosa, Joachim Spergser

**Affiliations:** 1 Department of Biological Sciences and Pathobiology, Centre of Pathobiology, University of Veterinary Medicine Vienna, Veterinaerplatz 1, 1210 Vienna, Austria; 2 Department of Biointeraction, Multidisciplinary Institute of Health, Federal University of Bahia, Vitória da Conquista, 40170-110 Brazil

**Keywords:** Antigenic phase variation, comparative genomics, Vpmas, surface lipoproteins, Mycoplasma pathogenicity, phylogenetics, GM139

## Abstract

*Mycoplasma agalactiae* causes one of the most serious forms of mycoplasmosis in small ruminants that is notifiable to the World Organization for Animal Health (WOAH)*.* Possessing a plastic genome, its Vpma and other surface antigenic variations play important roles in its pathogenesis and systemic spread within the goat or sheep host, as well as its ability to jump to wild animals. The Vpma phenotypic profile of strain GM139 was recently compared to that of the type strain PG2, whereby GM139 predominantly exhibited stable expression of a single VpmaV protein in comparison with the high-frequency variable expression of all six Vpma proteins in PG2. The complete genome sequence of GM139 was generated, annotated for detailed analysis of the *vpma* locus and compared with the finished genomes of three distinct *M. agalactiae* strains (PG2, 5632, and GrTh01). Interestingly, GM139 presented a longer distinct *vpma* locus with ten genes, one of which is a chimera between the *vpmaV* and *vpmaZ* genes of PG2*,* which correlates very well with previous immunoblotting results and was confirmed here by nanoLC-MS/MS analysis; five *vpmas* are completely unique, whereas the other four share similarities with the *vpmas* of 5632, one of which is also partially homologous to *vpmaZ*_*PG2*_. Additionally, features such as a larger *spma* locus, an intact *gsmA* known to encode a phase-variable glucan affecting serum resistance, and the presence of integrative and conjugative element (ICE) and transposases might have also influenced the pathogenicity and host range of these strains, segregating them into two well-separated phylogenetic clusters on the basis of a newly developed cgMLST scheme. This study highlights the plasticity and dynamic evolution of the *M. agalactiae* genome, especially its surface antigenic architecture.

**Supplementary Information:**

The online version contains supplementary material available at 10.1186/s13567-025-01531-x.

## Introduction

*Mycoplasma agalactiae* is one of the most significant pathogens of small ruminants and the main cause of contagious agalactia (CA) syndrome, which is notifiable to the World Organization for Animal Health (WOAH, founded as OIE) [1]. Although it is found worldwide, economic losses and social impacts are particularly compelling in regions where sheep and goat farming are important for livelihood, for instance, in the Mediterranean countries such as Spain, Italy, and Greece [2–5], as well as parts of Asia and South America [6, 7]. Once endemic, it is difficult to eliminate this pathogen because antibiotics, if effective, tend to promote the carrier state and recurrent infections. The available vaccines have several limitations and are mostly ineffective [4, 5, 8, 9]. Understanding its pathogenicity mechanisms is highly important for designing better anti-infectives and preventive measures.

*M. agalactiae* can serve as an ideal model to understand the pathogenicity and role of antigenic phase variation systems of mycoplasma pathogens, especially *Mycoplasma bovis*, which is economically even more devastating and shares close phylogenetic proximity, similar clinical signs, and many homologous gene families, including the *vsp* locus encoding phase-variable lipoproteins whose exact role is still unclear and represents a major knowledge gap in understanding its pathogenicity [10–14]. Additionally, it has one of the most plastic genomes that has undergone, and is still undergoing, massive genomic exchanges with other species, leading to modified pathogenicity attributes and spread to atypical newer hosts such as chamois and ibex [2, 15, 16]. In this context, the *vpma* locus encoding a family of variable surface lipoproteins is particularly well studied and constitutes the single known pathogenicity island among mycoplasmas [12, 17]. With few exceptions, field strains possess a single *vpma* locus, as in the type strain PG2, which contains six different but related *vpma* genes, with only one *vpma* gene expressed at a given time [18, 19]. Vpmas are immunodominant, abundantly expressed lipoproteins that vary at high frequency to evade the host’s immune response to cause persistent infections [12, 20]. Additionally, they serve as major cytadhesins leading to differential infection potential of Vpma expression variants, as witnessed during in vitro studies and experimental intramammary sheep infections [13, 21].

Considering the significant role of Vpmas in the host interactions and pathogenicity of *M. agalactiae*, the Vpma phenotypic profile of strain GM139 (goat isolate, USA) was compared with that of the type strain PG2 (sheep isolate, Spain) to study possible correlations between Vpma phase variability and the geographic distribution, animal host, and other pathogenicity traits of these strains. Interestingly, in this study, GM139 was observed to exhibit predominantly stable expression of a single VpmaV protein in comparison to the high-frequency variable expression of all six Vpma proteins in PG2. This was a peculiar case implying that GM139, although it expresses one of the most cytadhesive and invasive Vpma variants, might be severely compromised in its immune evasion capacity owing to its lack of visible phase variation in these immunodominant lipoproteins [22]. Furthermore, serum bactericidal assays to assess six individual Vpma phase-locked mutants also demonstrated significant differences between the PG2 and GM139 strains, with the latter showing remarkable resistance to sensitized and non-sensitized sheep sera [23].

GM139 genome sequencing and analysis were performed in this study to elucidate the genetic organization of the *vpma* locus in this strain and to understand the basis of the lack of expression of Vpmas other than the observed VpmaV expression on immunoblots. Apart from the detailed analysis of the *vpma* gene locus in GM139, comparisons were also made for the Vpmas and other surface lipoproteins and related elements contained within the genome of the type strain PG2 and two other distinct *M. agalactiae* strains expressing very different Vpma profiles, namely, strain 5632, with an expanded *vpma* repertoire of 23 *vpma* genes [18, 24], and GrTh01, with degenerated *vpma* loci [2]. Additionally, a core genome multilocus sequence typing (cgMLST) scheme was developed to assess the genomic relatedness and phylogeny of the above strains.

## Materials and methods

### Mycoplasma cultures and growth conditions

The *M. agalactiae* strains PG2 [25] and GM139 [26] were grown at 37 °C in modified SP4 broth for 2–3 days until a colour change occurred or on agar plates for 4–5 days as described previously [22].

### Genomic DNA extraction

For sequencing, a 15 mL culture of GM139 was centrifuged at 20 000 × *g* for 10 min, and DNA was extracted from the pellet using the DNeasy Blood and Tissue Kit (Qiagen). DNA quality, as well as quantity, was assessed with a NanoDrop™ 2000 (Thermo Scientific).

### Genome sequencing, assembly, annotation, and analysis

The genome sequence of GM139 was generated by combining Nanopore (MinION, Oxford Nanopore Technologies) and Illumina (MiSeq, Illumina) sequencing. Nanopore long reads, along with Illumina short reads, were hybrid assembled with the Unicycler pipeline [27], resulting in a circularized genome, which was then annotated utilizing the NCBI Prokaryotic Genome Annotation Pipeline (PGAP). Detailed analysis of the *vpma* locus and comparisons with the finished genomes of three *M. agalactiae* strains (PG2, 5632, and GrTh01) were performed using progressive Mauve alignment, which is available as a subprogram in Geneious Prime 2022.1 (Biomatters Ltd.).

### Triton X-114 phase partitioning and western blotting

Mycoplasma proteins were extracted using Triton X-114 (Sigma‒Aldrich, Austria), whereby the amphiphilic membrane proteins were partitioned into the detergent phase, the cytoplasmic proteins were partitioned into the aqueous phase, and the cytoskeleton proteins were partitioned into the insoluble phase [28]. Protein concentrations were measured with a NanoDrop 2000 spectrophotometer (Thermo Fisher Scientific, USA) using different dilutions of an albumin standard (2 mg/mL; Thermo Fisher Scientific) made in PBS (Gibco Life Technologies, USA) containing 10% phenylmethylsulphonyl fluoride (PMSF).

To assess the Vpma antigenic phenotype, the above three phases were subjected to SDS‒PAGE, and the separated proteins were transferred to Amersham™ Protran^®^ membranes (GE Healthcare Life Sciences, Carls Roth Germany). Immunostaining was carried out overnight at 4 °C with rabbit VpmaV-specific polyclonal antibodies, followed by incubation for 1–2 h at room temperature with swine anti-rabbit immunoglobulins/HRP (DakoCytomation, Denmark) before the blots were developed with 4-chloro-1-naphthol (Bio-Rad laboratories, USA) as described previously [19].

### Identification of proteins by nanoLC‒MS/MS analysis

To identify the Vpma protein expressed in the GM139 strain, SDS‒PAGE was run, and the gel was cut into two equal halves, each carrying the same set of samples. One half of the gel was used for western blotting as described in the above section, and the other half was stained with Coomassie Brilliant Blue R-250 for half an hour on a shaker, destained and subsequently imaged with ChemiDoc MP (Bio-Rad, USA). The gel bands corresponding to those recognized in the Western immunoblots were removed for in-gel digests.

Each excised band was split into smaller cubes and rinsed with 100 µL of water with gentle vortexing. Subsequently, 100 µL of aqueous ammonium bicarbonate (ABC; 100 Mm; Fluka Analytical, USA) was added before ultrasonication for 5 min, and the supernatant was removed. The same process was repeated with 100 µL of ABC and HPLC-grade absolute ethanol (Thermo Fisher Scientific) in equal amounts. The above ultrasonication procedure was repeated until the blue colour of the Coomassie blue stain disappeared. After further washing with water, ABC and acetonitrile hypergrade for LC/MC (Merck, DE), the gel cubes were dehydrated in a vacuum concentrator (Eppendorf, DE) for 10 min at RT. Disulphide bonds were reduced by rehydration in 50 µL of DL-dithiothreitol (DTT, 10 mM; Sigma‒Aldrich, USA) at 56 °C for 1 h at 550 rpm. Afterwards, DTT was removed, and iodoacetamide (55 mM; Sigma‒Aldrich, USA) was added for alkylation for 45 min at RT in the dark [29]. The sample was washed twice with ABC and once with acetonitrile, each step followed by ultrasonication and removal of the solution. After drying for 10 min in the vacuum concentrator, the proteins were digested either with Trypsin Gold: working solution composed of 120 µL of water, 120 µL of ABC and 10 µL of aqueous CaCl_2_ (120 mM; Sigma‒Aldrich, USA) added to 5 µL of trypsin stock (Trypsin Gold, mass spectrometry grade, Promega, Madison, WI, USA) or Trypsin/Lys-C Mix (mass spectrometry grade, Promega). The gel cubes were rehydrated in working solution for 20 min at 4 °C, the supernatant was discarded, 30 µL of ABC (50 mM, pH 8.5) was added, and the proteins were digested for 8 h at 37 °C on a thermomixer (550 rpm) and cooled to 4 °C [30]. For peptide extraction, the supernatant was transferred to a new tube, and 30 µL of acetonitrile:water:trifluoroacetic acid Optima LC/MS (50:45:5; Thermo Fisher Scientific) was added and incubated in an ultrasonic bath for 10 min. The supernatant in the peptide collection tube was then treated two more times with the same steps before vacuum concentration for 2.5 h at 45 °C and dissolved in 8 µL of 0.1% trifluoroacetic acid before analysis.

A nano-HPLC Ultimate 3000 RSLC system (Dionex) was used for high-performance liquid chromatography. The sample was desalted and preconcentrated with a 5 mm Acclaim PepMap µ-Precolumn (300 µm inner diameter, 5 µm particle size and 10 Å pore size; Dionex). The solution was loaded with 2% acetonitrile in ultrapure water (Optima LC/MS; Thermo Fisher Scientific) with 0.05% trifluoroacetic acid as the mobile phase at a flow rate of 5 µL/min. The analytical separation of peptides was performed on a 25 cm Acclaim PepMap C18 column (75 µm inner diameter, 2 µm particle size and 100 Å pore size) with a flow rate of 300 nl/min. For elution from the column, a gradient of 4% solution B [80% acetonitrile with 0.08% formic acid Optima LC/MS (Thermo Fisher Scientific)] was used for the first 7 min, which increased from 4 to 31% in the following 30 min and then to 44% in the last 5 min. Solution A consisted of ultrapure water with 0.1% formic acid. Finally, the column was washed with 95% solution B. The HPLC system was coupled to a high-resolution Q Exactive HF Orbitrap mass spectrometer via a nanoelectrospray ion source. Mass spectrometric data acquisition was performed in the mass range of m/z 350–2000 Da, with a resolution of 60 000, a maximum injection time of 50 ms and automatic gain control of 3 × 10^6^. The ten most intense ions were further fragmented by Orbitrap via high-energy collision dissociation activation over a m/z range of 200–2000, with a resolution of 15 000 (intensity threshold at 4 × 10^3^). Ions charged + 1, + 7, + 8 and greater than + 8 were omitted. The collision energy was set to 28. The automatic gain control was set to 5 × 10^4^ for each scan and the maximum injection time to 50 ms. To inhibit repeated peak fragmentation, the precursor ion masses were dynamically excluded over a time range of 30 s. The resulting spectra were analysed with Proteome Discoverer Software 2.4.0.305 (Thermo Fisher Scientific) and compared to the UniProt database and to the gene sequence of GM139 after transformation to an amino acid sequence via BLAST to identify the expressed Vpma.

### Core genome multilocus sequence typing (cgMLST)

A cgMLST scheme for *M. agalactiae* was developed employing Ridom SeqSphere + software (Ridom^©^ GmbH) as described previously for *M. hyosynoviae* [31]. Briefly, the reference genome (type strain PG2, acc. no. CU179680), and seven query genomes (strain GM139, acc. no. CP102095; strain GrTh01, CP039447; strain 5632, FP671138; strain 14668, JAGJTN000000000; strain 7784, SOSH00000000; strain 4055, JAGJTR000000000; strain 4867, SPQY00000000) were utilized to define the target genes of the cgMLST scheme. First, a target gene set suitable for cgMLST was filtered from the reference genome using the Target Definer tool. The seven query genomes were subsequently blasted against the reference genome’s target gene set to select shared targets (100% overlap, sequence identities >90%) constituting the final targets of the cgMLST scheme. Among the 21 *M. agalactiae* genomes available in the NCBI genome database, two have no reference genome sequences and are omitted by the NCBI staff owing to quality issues. The remaining 19 NCBI strains with qualified genomes, together with two unreleased in-house genomes of Mongolian *M. agalactiae* strains (OB564, UBS14), were typed by the newly developed cgMLST scheme to define individual allelic profiles, which were then utilized to construct a phylogenetic tree using the neighbor joining algorithm implemented in SeqSphere + software.

## Results

### Mass spectrometry analysis of GM139 Vpma

An earlier study revealed that GM139 expresses only VpmaV, and none of the other five Vpmas when tested by western blot and colony immunoblots using the Vpma-specific antibodies raised against each of the six Vpmas of the type strain PG2 [22]. This was a particularly surprising conformation, as the lack of other Vpmas, especially no phase variation, implied a compromised immune evasion capacity, as it is known to play an important role in *M. agalactiae* pathogenicity [13]. Genome sequencing of GM139 was undertaken to understand the basis of this peculiar phenotype and to decipher the genetic organization of the *vpma* gene locus. Although the GM139 *vpma* locus did not reveal the presence of a typical *vpmaV* gene as present in the PG2 strain, thorough analysis using the α-Vpma V_PG2_ Ab binding site revealed a match in the genome of GM139 with peg.754 vpma, which was confirmed to be a chimera between the VpmaZ and VpmaV proteins of PG2. To confirm this, Coomassie-stained gel bands corresponding to the α-Vpma V_PG2_ Ab-positive bands in the western blots were excised and subjected to nanoLC-MS/MS analysis (Additional file 1). Indeed, 754 vpma was identified in all 3 analysed positive bands with the highest number of peptides and highest sequence coverage observed in the band running parallel to the 43 kDa VpmaV-positive band of PG2 (Additional file 1), which used to be the only predominant α-Vpma V_PG2_ Ab-positive band in GM139 western blots during our earlier studies and was observed to show the same strong intensity as the PG2 band [22]. Further in-depth analysis of the *vpma* locus and details of all included genes were performed, and comparisons were made with the complete genomes of the type strains PG2, strain 5632 and strain GrTh01, as described below.

### General genome characteristics of strain GM139

**Figure 1:**
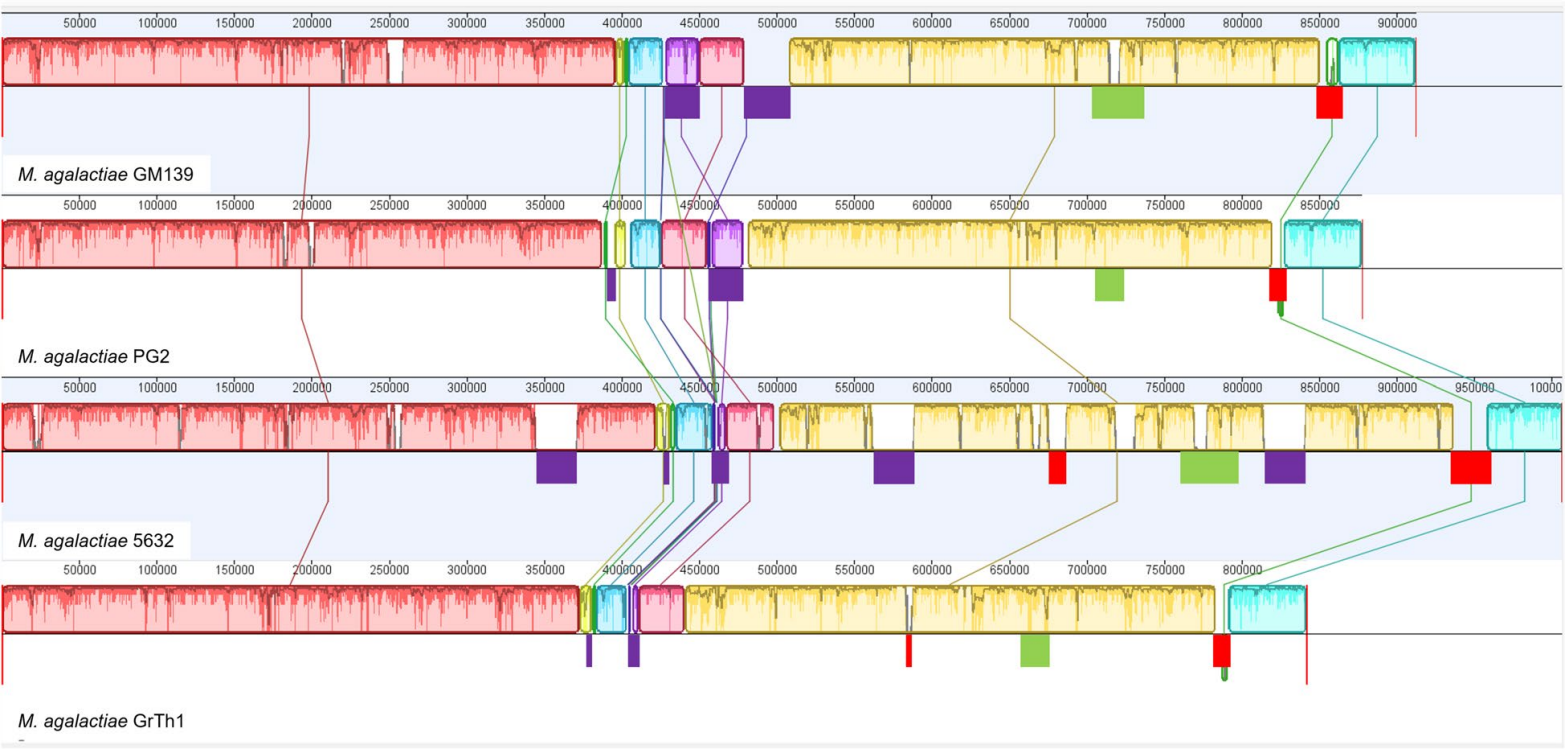
**Progressive Mauve alignment between the genomes of** ***M. agalactiae*** **GM139, PG2, 5632 and GrTh1**. Identical coloured, locally collinear blocks (LCB) represent homologous genomic regions shared between strains without sequence rearrangements. The red, green and purple boxes below the Mauve alignments illustrate the *vpma*, *spma* and ICE, respectively.

Hybrid assembly of Nanopore long reads and paired Illumina short reads resulted in a complete circular genome of 912,394 bp for *M. agalactiae* strain GM139 (CP102095). Overall, the genome arrangement was very similar to that of PG2 and other *M. agalactiae* strains, with no inversions observed, as shown in Figure 1. With 756 coding DNA sequences (CDSs), 34 tRNA genes and a G + C content of 29.7% most of the genomic characteristics were similar to those of the other strains except that GM139 possesses only one rRNA set of 16S and 23S, whereas   the other three strains contain  two full sets of rRNAs (Table 1). Interestingly, it harbours one copy of a 27-kb integrative and conjugative element (ICE) that is present in 5632 in three copies and a 21-kb ICE vestige similar to that of PG2. Considering other mobile genetic elements, the genome contains a relatively lower number of transposases (4 + 1 pseudo), especially in contrast to 5632 (15 + 2 pseudo). Plasmids or prophages were not detected.

**Table 1:** **General characteristics of GM139 compared with those of the** ***M. agalactiae*** ***strains*** **PG2, 5632, and GrTh01**.

	GM139	PG2	5632	GrTh01
Genome size (bp)	912,394	877,438	1,006,702	841,635
G + C (%)	29.7	29.7	29.6	29.8
CDS (with protein)	756	764	833	740
rRNA (5S, 16S, 23S)	2, 1, 1	2, 2, 2	2, 2, 2	2, 2, 2
tRNA	34	34	34	34
GenBank acc	CP102095	CU179680	FP671138	CP039447
Pseudogenes	10	5	2	22
ICE numbers	1 (+ 1 vestigial)	0 (+ 2 vestigial)	3 (+ 2 vestigial)	0 (+ 2 vestigial)
Transposases	4 (+ 1 pseudo)	1 (+ 2 pseudo)	15 (+ 2 pseudo)	0 (+ 2 pseudo)

### *vpma* gene locus

**Figure 2:**
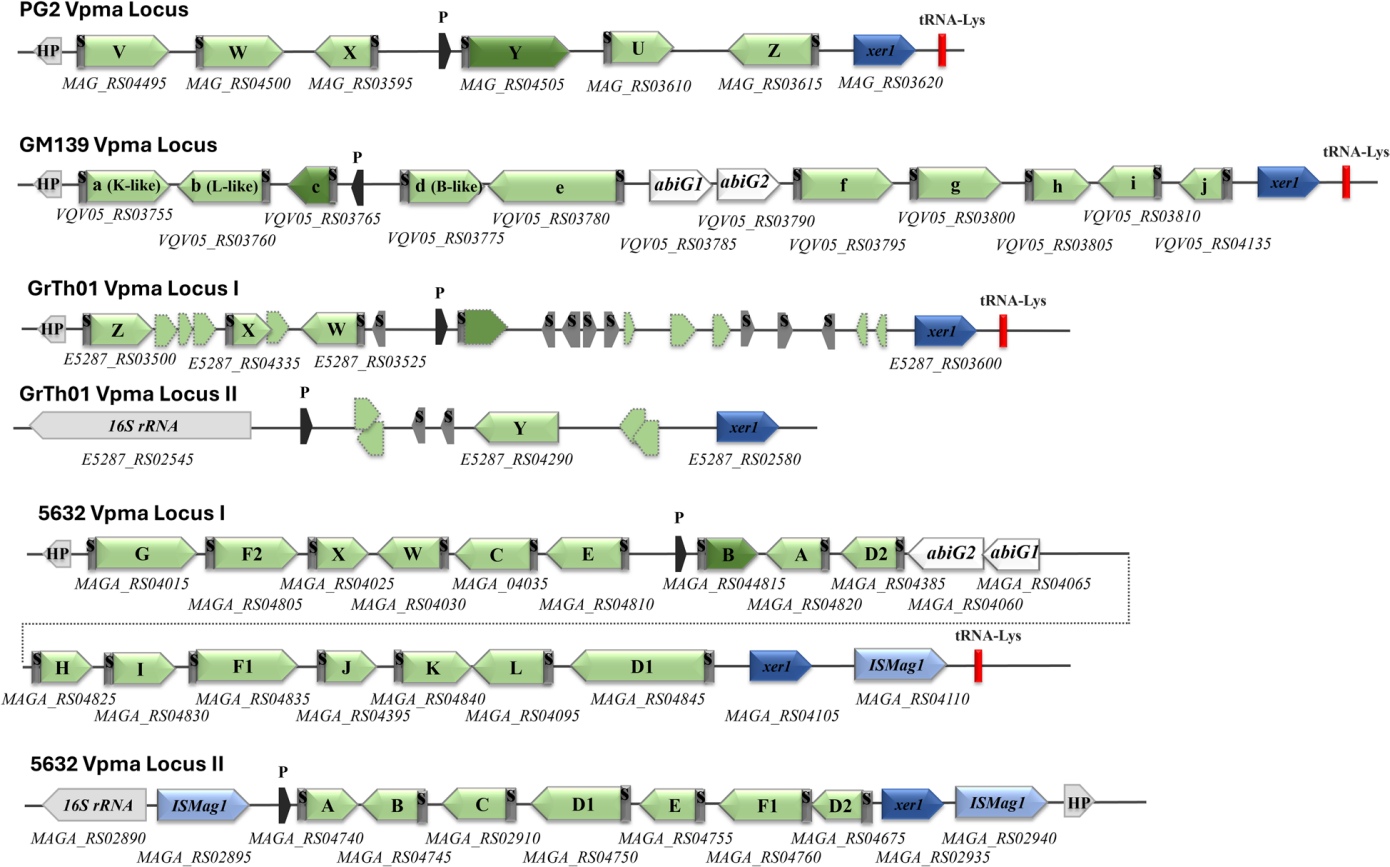
**Comparison of the Vpma loci of strain GM139 with those of the type strains PG2, 5632 and GrTh1.** Boxed arrowheads represent open reading frames (green: *vpmas*; blue: site-specific recombinase *xer1*; light blue: IS element; white: non-*vpma* related *abiG1* and *abiG2*). The expressed *vpma* gene downstream of the single promoter (P) in each locus is shown in a darker shade. S represents the signal sequence, HP represents a hypothetical protein, and red bars represent tRNA-Lys. Accession numbers/gene locus tags are inserted below each gene, and letters in parentheses refer to homologous genes in strain 5632.

**Figure 3:**
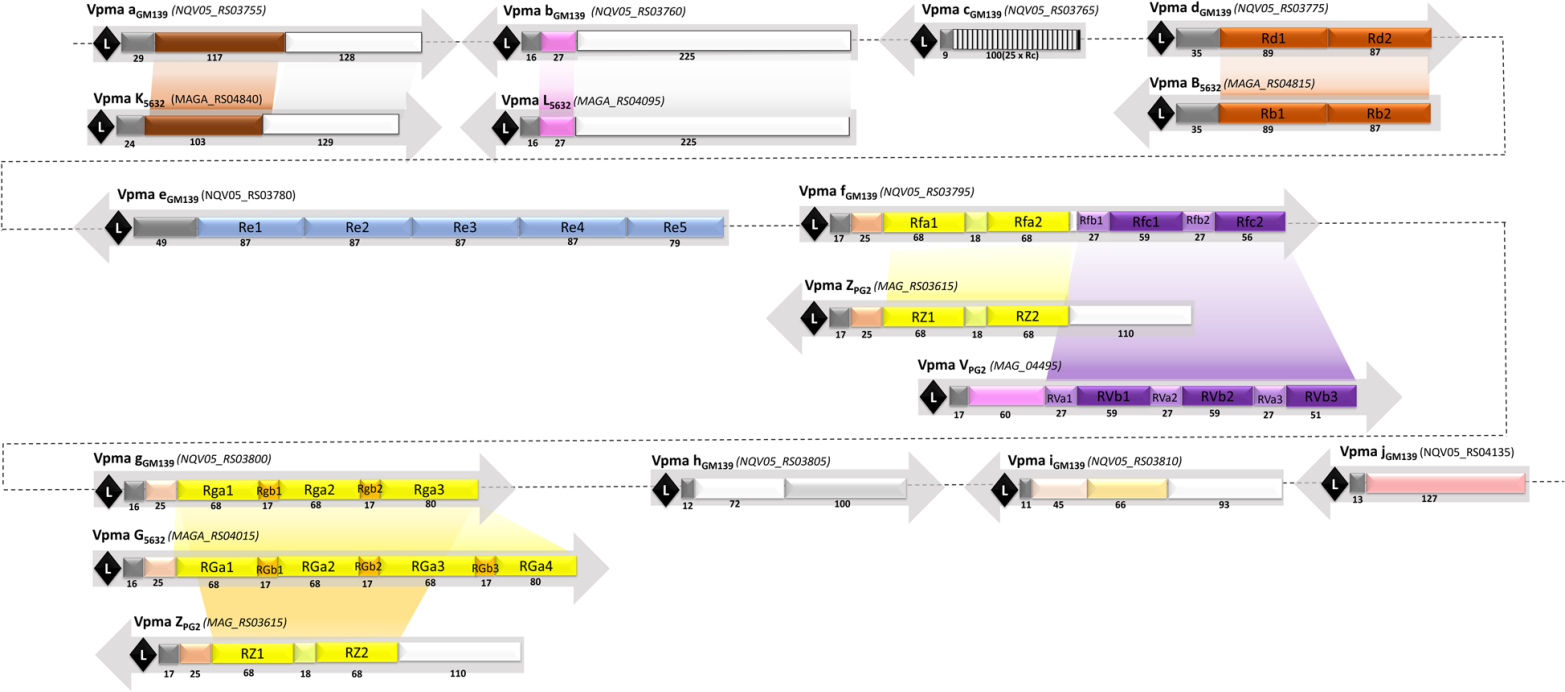
**Schematic of Vpma ORFs in strain GM139 with homologous regions in the 5632 and PG2 strains.** The conserved 25-aa leader sequence (L) is marked with black diamonds, homologous regions between the genes are depicted in the same colour when the aa identity is greater than 30%, and regions that are repeated within the gene are represented by R, followed by gene designation. The number of amino acids is indicated below each box. Comparisons are made with the structure of the *vpma* genes in strains 5632 and PG2 following the same colour pattern. Accession numbers/gene locus tags are inserted beside each gene.

Vpmas are the abundantly expressed immunodominant surface lipoproteins of *M. agalactiae* that vary at a very high frequency [12]. They play a significant role in pathogenicity by acting as major cytadhesins and immune evasion proteins [13, 20, 21]. Unlike 5632 and GrTh01, and as in the type strain PG2, there is a single *vpma* locus present in GM139 that presents all the typical features of a pathogenicity island, including the tRNA-Lys gene and a *xer1* gene encoding the site-specific recombinase that acts on the recognition sequences in the conserved 5’UTR of the *vpma* genes to cause Vpma phase variations by altering the *vpma* gene placed downstream of the single promoter [12]. The *vpma* locus of GM139 is approximately 13 kb long and contains a total of 10 *vpma* genes (Figure 2), among which four, namely, *vpma a*_*GM139*_*, vpma b*_*GM139*_*, vpma d*_*GM139*_* and vpma g*_*GM139*_ are homologous to *vpma K*_*5632*_, *vpma L*_*5632,*_* vpma B*_*5632*_ and *vpma G*_*5632*_ of strain 5632, respectively (Figure 3). Among these , *vpma g*_*GM139*_ also shares partial homology with *vpma Z*_*PG2*_ of the type strain PG2. One *vpma* gene, *vpma f*_*GM139,*_ has homology to PG2 and is a chimera between the *vpma Z*_*PG2*_ and *vpma V*_*PG2*_ genes of the PG2 strain. This finding correlated very well with our previous immunoblotting data [22] as well as the current LC–MS results explained in the previous sections. Most importantly, the remaining five of the ten *vpma* genes of strain GM139, namely, *vpma c*_*GM139*_, *vpma e*_*GM139*_, *vpma h*_*GM139*_, *vpma i*_*GM139*_, and *vpma j*_*GM139*_, are completely unique (Figure 3). Another interesting feature is the presence of the two non-*vpma* related *abiG1* and *abiG2* genes encoding the type IV toxin-antitoxin (TA) system [32], as was also shown for one of the *vpma* loci of 5632 [18].

### Other gene families encoding surface proteins

In the absence of a cell wall, the presence of several different repertoires of surface proteins in minimal mycoplasmas is suggestive of their important role in host cell interactions and pathogenicity. The *spma* locus, encoding a family of surface lipoproteins of unknown function, is approximately 24.852 bp in length in GM139, which is similar to 5632. On the other hand, both PG2 and GrTh01 have a considerably shorter *spma* locus of 14 kb. The 5ʹ untranslated region of each *spma* gene carries a polyG tract that is often indicative of phase-variable expression in mycoplasmas via the control of downstream transcription [24]. Another family of surface proteins with unknown function is the Bacteroides-like surface protein A (BspA) family, which bears the DUF285 motif. Eleven full copies of *bspA* are present in GM139, a count that is again quite similar to the 12 copies in 5632, whereas GrTh01 harbors only four entire and four pseudogene *bspA* [2].

### Phase variable capsular polysaccharide and important pathogenicity determinants

In addition to surface proteins, *M. agalactiae* is also known to secrete a capsular polysaccharide that undergoes high-frequency ON/OFF phase variation due to changes in the length of a poly(G) tract located in the *gsmA* gene.

**Figure 4:**
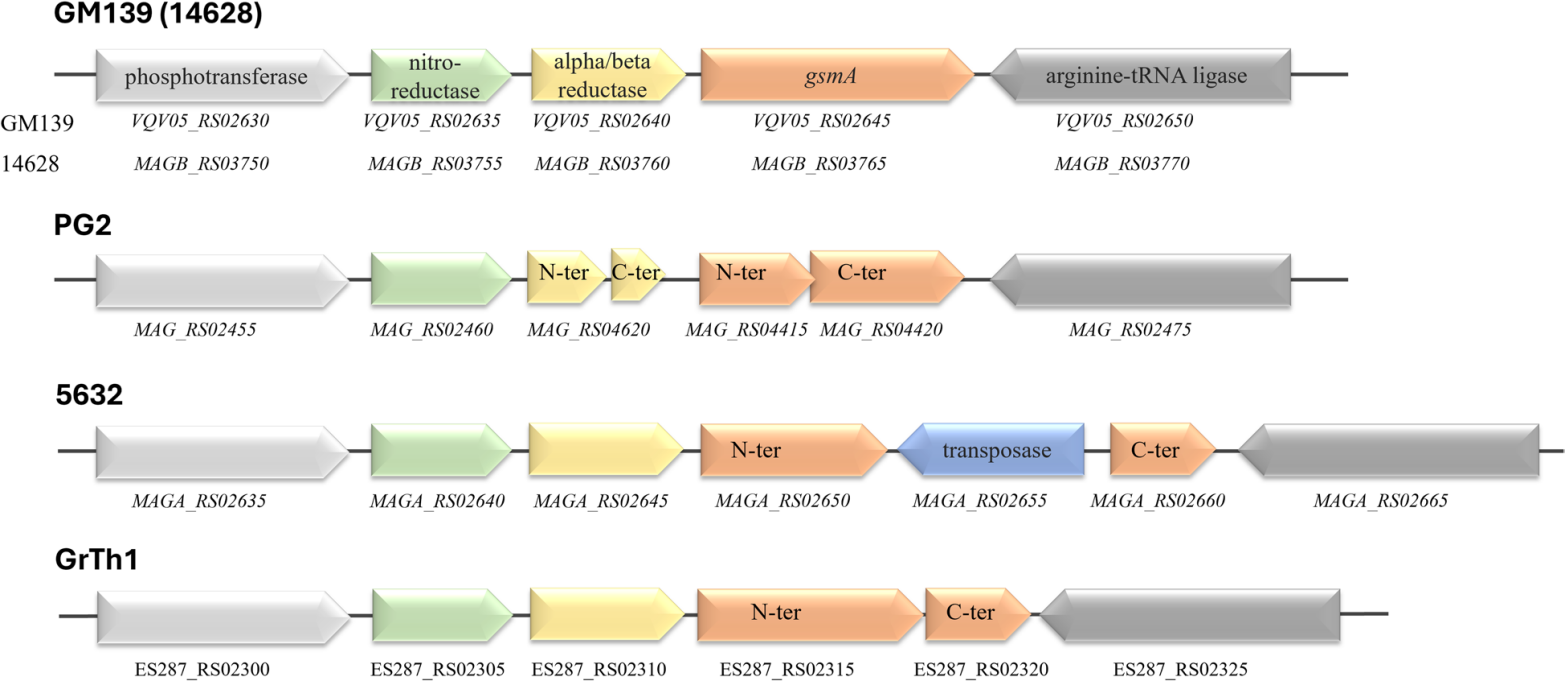
**The** ***gsmA*** **gene encoding β-(1 → 6)-glucopyranose in** ***M. agalactiae*** **strain 14628 is intact in GM139**. Comparisons are shown with truncated copies of the *gsmA* gene in all of the compared strains, i.e., the type strain PG2, and strains 5632 and GrTh1. Accession numbers/gene locus tags are inserted below each gene.

The secretion of β-(1 → 6)-glucopyranose was shown to be dependent on the presence of a functional *gsmA* gene in strain 14628 and increased the susceptibility of the pathogen to serum killing [33]. Similarly, GM139 also carries a functional *gsmA* gene, as observed in strain 14628, whereas it is truncated in strains 5632, PG2 and GrTh01 (Figure 4). Furthermore, full copies of other known virulence genes, such as the *nifS–nifU*, and those encoding the adhesin P40 and P80 lipoproteins, were found in GM139 [2].

### Core genome MLST

**Figure 5:**
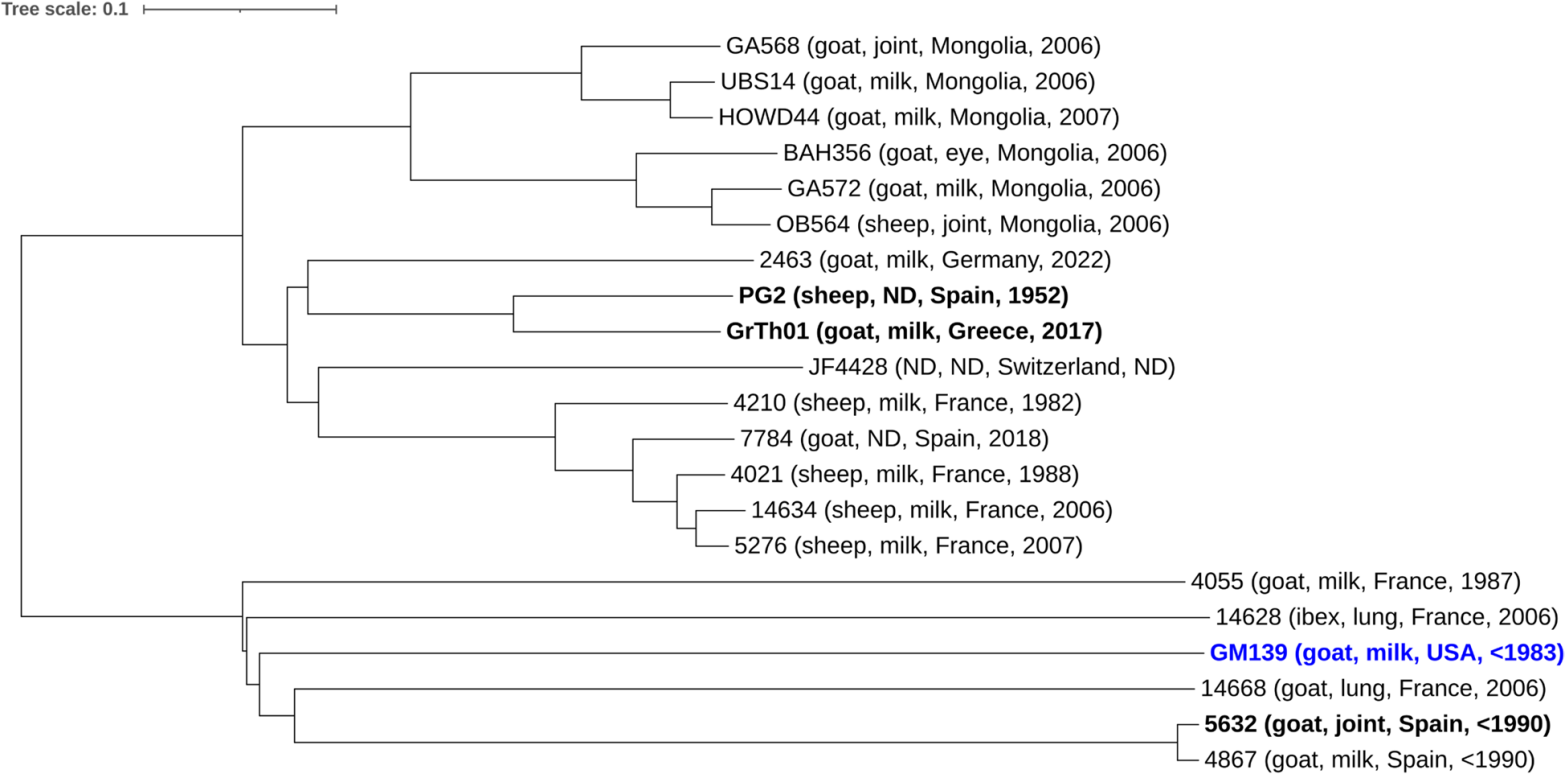
**CgMLST of** ***M. agalactiae*** ***strains***. Phylogenetic tree based on allelic profiles of 476 core genome targets from 21 *M. agalactiae* strains, including all the qualified *M. agalactiae* genomes available at NCBI and two in-house genomes (OB564 and UBS14). The tree was constructed using the neighbour-joining algorithm and the pairwise ignoring-missing-values option in SeqSphere +. Information on the origin of the strains is provided in parentheses (host, sample type, country, year of isolation; ND not defined).

From the reference genome (PG2) and 7 query genomes (GM139, GrTh01, 5632, 14668, 7784, 4055, 4867), a cgMLST scheme with 476 target genes (506,871 bases) was defined, covering 58% of the reference genome sequence. To gain insights into the population structure of *M. agalactiae* and to evaluate the genome-wide relatedness of the strains included in comparative genomics, the newly developed cgMLST scheme was applied to 21 strains, which includes all the qualified *M. agalactiae* genomes available at NCBI, as well as two unreleased in-house genomes. This resulted in a clear separation of the *M. agalactiae* strains into two main clusters (Figure 5). The larger cluster comprises 15 *M. agalactiae* strains isolated from sheep and goats in Asia (Mongolia) and Europe, including the type strain PG2 and its closest relative, GrTh01. The smaller cluster (six *M. agalactiae* strains) contains only strains isolated from caprine hosts (goat, ibex) and illustrates a closer phylogenetic relationship between GM139 and 5632.

## Discussion

In addition to their small size and minimal genomes, bacteria belonging to the genus *Mycoplasma* are successful pathogens that cause persistent chronic infections that are difficult to cure. Much of this is attributed to their intelligent strategies to overcome host immune responses and to survive and adapt amidst changing host environments and antibiotic pressure [34–36]. The presence of large repertoires of multigene families encoding surface antigens that undergo high-frequency phase variations is one such tool that many *Mycoplasma* pathogens use for immune evasion and host survival [34, 37]. The Vpma family of surface lipoproteins of *M. agalactiae* is one such antigenic phase variation system that has been very well studied and is perhaps the only one in this field for which “phase-locked” mutants were constructed to study the role of individual Vpma expression variants in the intramammary sheep infection model as well as several in vitro assays [12, 18, 19]. Not only is Vpma phase variation imperative for host immune evasion, survival and persistence of the pathogen, but Vpma expression variants also demonstrate differential infection potential, which correlates very well with their relative adhesion and colonization capacity [13, 20, 21]. Considering the significance of Vpmas and their antigenic variation, it was surprising to observe the expression of a single VpmaV variant in  immunoblots of strain GM139 [22]. Although VpmaV demonstrates one of the highest adhesion and invasion capabilities among the six Vpma variants of the PG2-type strain [21], the lack of expression of the other five variants in GM139 could imply a weakened immune evasion capacity for this strain and hence compromised survival and reduced pathogenicity potential. However, even though there are no published reports of any systemic infections with GM139, it is known to cause mastitis in goats [26]. Moreover, in an earlier study, its molecular analysis had revealed a completely different *vpma* hybridization pattern than that of PG2 and eleven other tested *M. agalactiae* clinical isolates from Israel [38]. For these reasons, we were interested in deciphering its *vpma* gene locus to understand its Vpma expression profile, and in the process also to compare its Vpma and other related surface proteins with those of three other *M. agalactiae* strains, namely, PG2, 5632 and GrTh01.

Genome analysis of GM139 revealed an unprecedented Vpma profile, with five of the ten *vpma* genes being completely unique, with no known homologies with any *vpma* genes available at GenBank, including those present in the well-studied PG2, 5632 and GrTh01 strains. Among the remaining five genes, three have homology with *vpma* genes of 5632, one has homology to 5632 as well as PG2, and of particular interest to us, one of the GM139 genes, *vpma f*_*GM139,*_ is a chimera of *vpma Z* and *vpma V* of PG2 (Figure 3). This was also confirmed by LC/MS analysis of the Western blot bands, whereby VpmaV_PG2_-specific antibodies recognized the homologous region in GM139 (Additional file 1). Interestingly, as observed in 5632, the GM139 *vpma* locus also harbors the *abiG1* and *abiG2* genes, which are thought to have been transferred from *Streptococcus agalactiae* via horizontal gene transfer (HGT) [18]. This gene pair has recently been described as a putative type IV TA system that likely contributes to the pathogenicity and persistence of mycoplasmas and exacerbates the buildup of antimicrobial resistance [32]. Found in strains of *M. agalactiae* and *M. feriruminatoris*, the protein signatures of this pair have been restricted only to antitoxins [18, 32], although PCR products of both genes were obtained in approximately 11% of the tested *M. agalactiae* strains belonging to very different geographical origins [18]. Nevertheless, as in most TA systems carrying signatures of HGT, the presence of *abiG1*/*abiG2* in the *vpma* loci assigned as “genomic islands” by predictive software tools [17] is interesting. Similar variably expressed surface lipoproteins in avian mycoplasmas, namely, *M. gallisepticum*, *M. imitans* and *M. synoviae,* are encoded by large multigene families that also appear to have resulted from HGT [39]. Moreover, involved in host colonization and immune evasion, the *vpma* locus bears all the characteristics of a typical pathogenicity island [12] and might serve as a hotspot for *abiG1*/*abiG2* insertions, which perhaps later got  lost in several *M. agalactiae* strains because of deletions caused by frequent DNA recombination events that are common within the locus [18]. Indeed, cgMLST analysis and comparison with some of our in-house available *M. agalactiae* strains revealed that GM139 was close to 5632, with both strains carrying the *abiG1/abiG2* genes, whereas PG2 and GrTh01 lacking these genes are positioned further apart (Figure 5).

Another similarity between the GM139 and 5632 strains with respect to genes devoted to phase-variable surface proteins is observed for the *spma* family. This locus is approximately 25 kb long in both 5632 and GM139, unlike the PG2 and GrTh01 strains, where it is reduced to almost half the size (14 kb). Although not detected in the proteome of 5632, the locus is composed of several putative CDSs encoding similar signal peptides, conserved lipoboxes followed by repeats of amino acid motifs, and polyG tracts in the 5’ untranslated regions of each putative *spma* gene, all of which point towards their phase variable surface expression [24]. As these genes have been exchanged with members of the “mycoides” cluster, they are likely to play an important role and are perhaps expressed during specific stages of infection only. In contrast, *spma* genes present on a much-reduced locus in the PG2 strain do not have any orthologues in the “mycoides” cluster [24]. Notably, the repertoires of gene families encoding surface variable proteins are significantly curtailed in strain GrTh01, which has been correlated with its limited capacity to cause a rather mild disease and that too only  in goats found in a mixed herd of sheep and goats [2]. Compared with the 5632 strain, which has two *vpma* loci, each with its own promoter, and carrying seven and 16 *vpma* genes, respectively, leading to the concomitant expression of two Vpmas at a given time, GrTh01 possesses two degenerate *vpma* loci carrying *vpmaW, vpmaX* and *vpmaZ* (at locus 1) and *vpmaY* (at locus 2), while the rest of them are pseudotruncated genes. If at all any Vpma is expressed in GrTh01 is doubtful, as none of the entire *vpma* genes are found directly downstream of the known *vpma* promoter sequences. This reduced *vpma* gene set, especially the lack of *vpmaV* in GrTh01, is considered one of the main deciding factors for the reduced host range (only Caprinae) and symptoms limited to the mammary glands [2].

In addition to the minimal *vpma* and *spma* loci, the *bspA* gene family is also considerably reduced in GrTh01, with only 4 entire *bspA* genes, in contrast to the 11, 9 and 12 *bspA* genes present in the GM139, PG2 and 5632 strains, respectively. BspA proteins, with homologous regions also present in *M. bovis*, are hypothetical surface lipoproteins with a DUF285 motif and are believed to be a part of the gene pool that underwent HGT with the “mycoides” cluster [2, 24]. DUF285 repeats have been identified among diverse bacterial and eukaryotic microbes [40] and are considered important for mycoplasma animal host classification [41]. Proteins carrying DUF285 domains are found in *M. bovis*, as well as in  several members of the *M. mycoides* cluster, including the phylogenetically close member *M. feriruminatoris,* where eight CDSs encoding these motifs are found on one of its ICE [15, 42]. Overall, the above data pertaining to variable surface proteins point towards GM139’s proximity to strain 5632 (as also witnessed in the cgMLST analysis) (Figure 5), which possesses one of the most elaborate and complex surface variable proteins [18, 24], and GM139 seems to be an intermediary between PG2 and 5632 in many aspects.

Furthermore, unlike 5632, PG2 and GrTh01, the presence of an intact *gsmA* gene in GM139 likely provides it an extra advantage in the host blood stream by switching ON/OFF the expression of a capsular polysaccharide that controls its serum-killing susceptibility, as shown for *M. agalactiae* strain 14628 [33], a strain that is phylogenetically related to GM139 (Figure 5). Compared with the PG2 strain and related mutants, GM139 exhibited increased resistance to serum killing in a previous study [23]. Interestingly, the secretion of this β-(1 → 6)-glucopyranose, a rare polysaccharide in prokaryotes, was also detected in *M. mycoides* subsp. *capri* PG3^T^, another pathogen of small ruminants [33]. Moreover, during an experimental infection study, goats infected with a mutant strain of *Mycoplasma mycoides* subsp. *capri* lacking the capsular polysaccharide presented only transient fever, indicating that it is an important pathogenicity determinant [43].

Inter- or intraspecies chromosomal exchanges in minimal mycoplasmas help them acquire new pathogenicity determinants via HGT and rely on the presence of mobile genetic elements such as ICE or insertion sequences (ISs). Mycoplasma ICEs are conjugative transposons that encode approximately 20 structural genes that are flanked by inverted repeats and help in the acquisition of small/large chromosomal fragments from any part of the donor, leading to mosaic genome progeny [17]. Strain 5632 is an exceptional strain and has one of the largest *M. agalactiae* genome sizes of approximately 1006.7 kb, whereby 76% of its additional genomic material is composed of mobile genetic elements compared with PG2 (877.4 kb), the latter being a prototype of the majority of *M. agalactiae* strains [18]. Compared with PG2, which lacks ICE and merely shows a single transposase, 5632 has 3 functional ICEs and 15 different transposases. Interestingly, strain GM139 also carries one functional copy of the 27 kb ICE and four transposases, which might have caused chromosomal exchanges, as evidenced by the presence of *abiG1/abiG2* genes within the *vpma* locus, which in itself has a distinct repertoire of *vpma* genes, half of which are unique, few are homologous to 5632, one has shared regions with both PG2 and 5632, and yet another one is a hybrid of two different PG2 *vpma* genes.  Laced with ICE, strain GM139 retains sexual competence for dynamic exchanges with other* Mycoplasma* species, thus increasing its pathogenicity potential. As such, HGT is quite prevalent in ruminant mycoplasmas and has likely aided in host jumps. For example, atypical *M. agalactiae* strains infecting wild ungulates, such as chamois and ibex, contain gene sequences closely related to those of *M. conjunctivae* [16, 44]. Interestingly, our cgMLST analysis revealed that *M. agalactiae* strains such as GM139 and 5632, which are well equipped with genomic “tools” for HGT and possess elaborate and more complex antigenic phase variation systems, belong to a phylogenetic cluster that contains only strains isolated from caprine hosts (goat, ibex) that are separated from the PG2-type strain (Figure 5). Notably, some commercial contagious agalactia vaccines are not as effective in goats as they are in sheep [4]. If this has anything to do in these cases with caprine *M. agalactiae* strains being genomically more complex, especially due to acquiring new virulence loci from other microbes via HGT is an interesting possibility although this needs to be investigated.

Overall, the GM139 genome sequence possesses a distinct *vpma* repertoire and several other lipoproteins and putative phase variable surface loci. Lipoproteins play significant roles in mycoplasma pathogenicity, ranging from nutrient acquisition to adherence and interaction with the host, as well as immune evasion and induction of host immune responses [20, 21, 39, 45–47]. Furthermore, this strain is also armed with the necessary tools, such as ICE and transposases, to undergo dynamic HGT to acquire new pathogenicity factors that could increase its host range and tissue tropism.

## Supplementary Information

**Additional file 1: Mass spectrometry analysis of GM139 Vpma.** SDS‒PAGE Coomassie (A) and western blot (B) analyses of the triton phase of *M. agalactiae* strains GM139 and PG2 using  α-VpmaV_PG2_ antibody. The 43-kDa Coomassie-stained band of GM139 corresponding to the strong positive band of PG2 in the western blot, also observed as the sole positive band during our earlier western blot study, as well as  the 14 kDa band and the strongly reacting gel front were excised and analysed by LC‒MS. (C) Peptides identified in the  43 kDa, 14 kDa and the running gel front of GM139 corresponding to peg.754 vpma after a database search in addition to the GM139 in-house database.

## Data Availability

The datasets generated during and/or analysed during the current study are available in the NCBI genome repository [48].
