# Supplementary material for: Comparative genomic analysis of Mycoplasma agalactiae strain GM139 highlights unique surface architecture and pathogenic determinants
Source: Vet Res. 2025 May 24;56:106. doi: 10.1186/s13567-025-01531-x (PMC12103780; doi:10.1186/s13567-025-01531-x)
Supplement: Supplementary file 1 — Additional file 1: Mass spectrometry analysis of GM139 Vpma. SDS‒PAGE Coomassie (A) and western blot (B) analyses of the triton phase of M. agalactiae strains GM139 and PG2 using α-VpmaVPG2 antibody. The 43-kDa Coomassie-stained band of GM139 corresponding to the strong positive band of PG2 in the western blot, also observed as the sole positive band during our earlier western blot study, as well as the 14 kDa band and the strongly reacting gel front were excised and analysed by LC‒MS. (C) Peptides identified in the 43 kDa, 14 kDa and the running gel front of GM139 corresponding to peg.754 vpma after a database search in addition to the GM139 in-house database. [file 13567_2025_1531_MOESM1_ESM.docx]

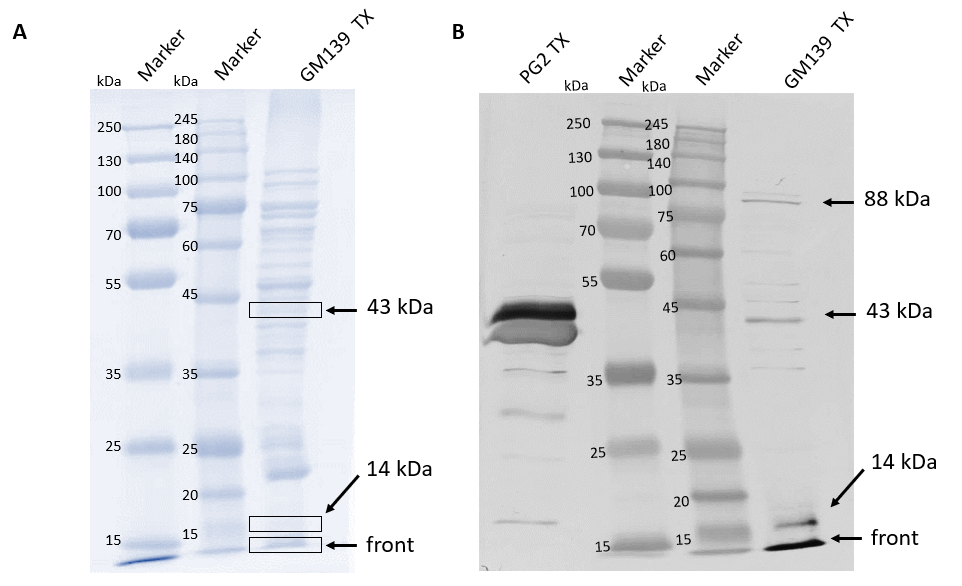


**C**

| **Accession** | **Score Sequest**  **HT** | **Coverage [%]** | **# Peptides** | **# PSMs** | **# Unique Peptides** |
| --- | --- | --- | --- | --- | --- |
| fig\|6666666.901569.peg.754vpma | 443.98 | 67.5 | 31 | 244 | 29 |
| fig\|6666666.901569.peg.754vpma | 15.47 | 19.0 | 7 | 8 | 5 |
| fig\|6666666.901569.peg.754vpma | 5.77 | 5.1 | 2 | 2 | 1 |
